# E‐Professionalism's Impact on Dental Professionalism: A Rapid Scoping Review

**DOI:** 10.1111/eje.13051

**Published:** 2024-10-31

**Authors:** Melanie Nasseripour, Angela Harkins, Patricia Neville, Amitha Ranauta

**Affiliations:** ^1^ Faculty of Dentistry, Oral and Cranio‐Facial Sciences, Centre for Oral, Clinical & Translational Sciences King's College London London UK; ^2^ University of Glasgow Dental School Glasgow UK; ^3^ Bristol Dental School Bristol UK; ^4^ Barts and the London School of Medicine and Dentistry Queen Mary University of London London UK

**Keywords:** digital social media, ethics, literature review, professionalism

## Abstract

**Background:**

Dental regulators and educational institutions are increasingly concerned about the influence of digital platforms used by the profession for social, business, digital interaction with the public/patients and its impact on the professionalism in practice now and going forward. However, academic knowledge and research within dentistry are relevant to e‐professionalism at a level of engagement and approach in delivering guidance to students through the current dental curriculum. The question therefore asked was what breadth of academic material, research, debate and discourse is available to inform our understanding, guidance and teaching on this ever‐evolving topic.

**Aim:**

To map how e‐professionalism has developed in academic dental literature as a topic within the study of professionalism in practice.

**Method:**

A rapid scoping review was conducted to identify published research that describes and tests the topic of professionalism from 2016 to 2023. Studies were synthesised narratively using thematic analysis to inform the understanding of what has been already researched in the field.

**Results:**

Thirteen articles were included in the review. After undertaking a thematic analysis, five themes were constructed. These included: curriculum, opportunities and safe professional use, reflections, personal and professional identity issues, and students as co‐creators.

**Conclusions:**

Within dental research there is consensus that e‐professionalism could present a tangible threat to the identity of dental professionals their clinical practice and interaction with patients/public however, less is known about what educational strategies are most effective when teaching e‐professionalism.

## 1 Introduction

Research on social media, its impact on society and professional practice is an expansive field of research. The research has generated a body of evidence which has fuelled persistent and contradictory opinions on the impact of social media and whether it is a force of social good or social harm in society. Research has already noted that social media has changed the perception of the oral health landscape, in terms of how social media frames oral health, access to services as well as the potential impact on professional reputation and the presentation of the dental profession to society [1, 2]. However, concerns about the perceived dangers of social media have grown. There is an increased awareness of the role of social media in misinformation, synthetic information, dis‐information and de‐information [3, 4]. Academic dentistry and dental education need to be aware of the evolution of the diverse opinions, debates and challenges in order to develop appropriate curricular innovations and research agendas. Writing back in 2011, Hobbs argued that all students need to develop competencies to deal with digital technology to better navigate the multiple and overlapping social, technical and ethical challenges associated with living in a networked society. Being digitally literate involves the promotion of critical thinking about how to use social media as a tool and its direct link with citizenship and civic culture [5].

Professionalism has become a standalone course in most of the Dental School and Dental Care partner school curricula over the past 20 years. Despite this, there is less than clear guidance on how best to teach and instruct on professionalism and even less on the teaching of e‐professionalism for dental students. To gauge the breadth and scope of this curriculum in professionalism, the ADEE Community of Practice in Professionalism embarked in 2022–2023 on two separate projects:

- the first looking at mapping the Professionalism curriculum across ADEE membership in conjunction with a literature review.
- the second looking specifically at the professionalism as applied to Digital Social Media and AI or what is sometimes coined as e‐professionalism in conjunction with case studies.

In this paper, we will outline the second project which aims to establish a position and propose on guidance on professionalism when using Digital and Social Media and AI. This paper seeks to illustrate the scope and scale of relevant research into social media and reveals the current state of evidence or literature available between (2016 to present) on this topic in dentistry. Literature from 4 databases was reviewed to identify types of research conducted on the topic of social media and its prominent themes. This will facilitate the identification of best practice and any voids in the literature which need to be addressed collectively as a Community of Practice.

## 2 Materials and Methods

This scoping review sought to identify how academic dentistry engaged with the topic e‐professionalism and to establish a position on the current evidence base within the discipline. In recognition of the unprecedented pace of digital technological innovation and the resulting ever‐changing composition of the field combined with the need from within the profession for better guidance on e‐professionalism, we decided to undertake a rapid review of the relevant literature, following the Cochrane Rapid Review model [6]. They define a rapid review as: ‘A rapid review is a form of knowledge synthesis that accelerates the process of conducting a traditional systematic review through streamlining or omitting specific methods to produce evidence for stakeholders in a resource‐efficient manner’ [7].

**FIGURE 1:**
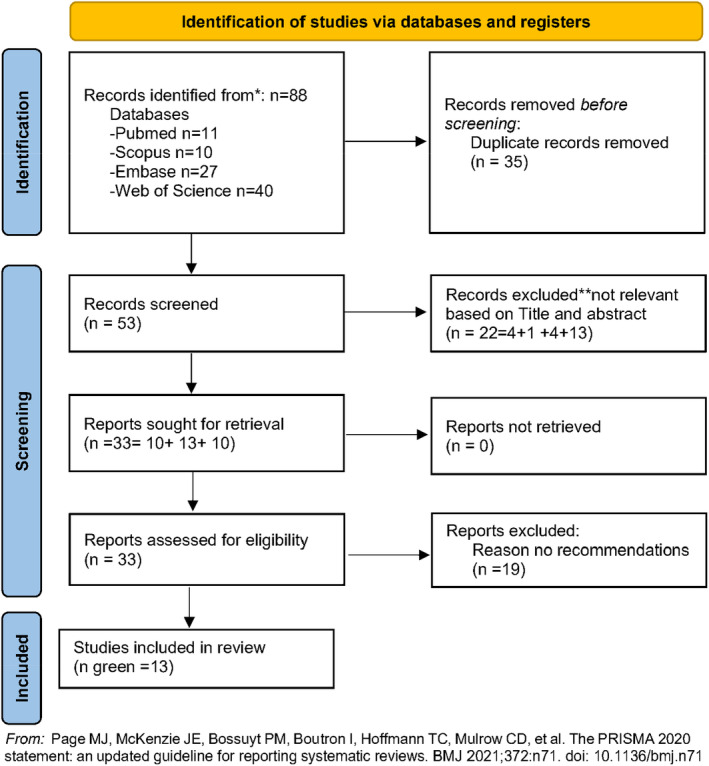
PRISMA flow chart.

The Cochrane Rapid Review model covers the following steps [6]:

1. *Setting the research question*: The parameters of the rapid review were informed by the objective of the study: to establish recommendations for use of technology (including social and digital media, AI, etc.) for teachers and UG students. The authors refer to the 2005 Royal College of Physicians definition, that professionalism is ‘a set of values behaviours and relationships that underpin the trust the public has in doctors’.
2. *Setting eligibility criteria*: We aimed to include peer‐reviewed journal articles across a range of study types, from systematic reviews, rapid reviews, RCTs, case studies, opinion papers. Grey literature and policy/guidance documents from relevant stakeholders, including universities, professional regulators, professional organisations, governmental departments would be included. All studies published in peer‐reviewed journals from 2016 to 2023 were included. As English was the primary language among the research team, only studies published in the English language were included.
3. *Searching the literature*: The search strategy was developed by the research team. The initial search string was developed and piloted to improve the sensitivity and specificity of the search. The final search string was as follows: (Dent* or nursing) and (Professionalism or e‐professionalism) and (digital media or social media or artificial intelligence or technology) and (English) and (2016 to present). Four databases were used in the scoping review—PubMed, Web of Science, Embase and Scopus. To make the scoping review feasible, the database searches were divided among the research team. Researcher MN reviewed the Web of Science, Researcher PN Embase and Scopus and Researcher AH searched PubMed. The search strategy is summarised in a PRISMA flow chart in Figure 1.
4. *Study selection*: Titles and abstracts were screened by all the reviewers. Any article that met the inclusion criteria progressed for full‐text screening. A colour‐coded (red exclude, brown for discussion, yellow and green for final inclusion) shared spreadsheet was developed to aid the screening process. The criteria for colour coding followed the inclusion/exclusion criteria. Where all three reviewers agreed the decision was upheld. Any disagreement was discussed by all three reviewers and resolved collegially.
5. *Data extraction*: The included studies so far were subjected to a full‐text review shared between the four researchers MN, PN, AH, AR. Each researcher then proceeded to data extract from the included studies, using a standardised data extraction Excel spreadsheet after a calibration session [6]. Same colour coding was used in this spreadsheet (red exclude, brown for discussion, yellow and green for final inclusion), taking into consideration also relevance to addressing the need for e‐professionalism curriculum guidance. The final included studies were thus determined.
6. *Risk of bias assessment*: Two of the reviewers (AR, MN) conducted a critical appraisal of the results using appropriate critical appraisal tools based on suitability for the type of study [8, 9, 10]. The key aspects and results of the appraisal of the studies included are summarised in Table 1.
7. *Synthesis*: The results of the rapid review were synthesised narratively by the team.

**TABLE 1:** Main characteristics of the studies included in the review and critical appraisal.

	Author and date	Country	Study design	Participant type	Key topics	Critical appraisal
1	Vukusic Rukavina T et al., 2021	Croatia	Review	NA	e‐Professionalism such as professional networking and collaboration, training and education	High
2	Kamarudin Y et al., 2022	Malaysia/Indonesia	Cross sectional	Dental students (undergraduate)	e‐Professionalism such as professional marketing, training and education	Medium
3	Guraya SS et al., 2022	Bahrain/UAE/Malaysia/Ireland	Mixed method	Medical students (undergraduate)	Teaching/pedagogy/DSM guidance	High
4	Gormley M et al., 2021	UK	Qualitative	Dental students (undergraduate)	Teaching/pedagogy/e‐professionalsim awareness	High
5	Kenny P et al., 2016	UK	Cross sectional	Dental students (undergraduate)	Teaching/risk management	Medium
6	Holden A, 2017	UK/Australia	Opinion	NA	Reputation/DSM guidance	High
7	Arayapisit T et al., 2021	Thailand	Descriptive	Dental students (undergraduate)	DSM/privacy	Medium
8	Knott PN et al., 2018	UK	Mixed method	Dental students (undergraduate)	DSM use	Low
9	Neville P et al., 2020	UK	Review	NA	Digital impact on practice	Medium
10	Uma E et al., 2021	Malaysia/Finland	Comparative	Dental students (undergraduate)	DSM use/teaching	High
11	Shah R et al., 2019	UK	Audit	Dental students (undergraduate) and dental educators	DSM/privacy	Medium
12	Kasperiuniene J et al., 2019	Lithuania	Review	NA	Professional identity	Medium
13	Khan MI et al., 2021	Australia	Quant non RCT	Healthcare professionals	Technology acceptance model (TAM)	Medium

## 3 Results

The aim of this rapid review was to establish from current research a position relating to Digital Social Media, Business Media and AI technologies on professionalism guidance or recommendation. Our literature review led to the inclusion of 13 manuscripts for synthesis and subsequent analysis. We noted a diverse collection of manuscripts where six of them explored undergraduate dental students [11, 12, 13, 14, 15, 16], one study surveyed postgraduate students [17] and five of them were reviews of which two were registered systematic reviews [18, 19, 20, 21, 22]. Of the studies within this collection, one was cross‐institutional but in the same country [14] (Kenny) and three studies were cross‐institutional across multiple countries [13, 16, 23], two studies compared dental students and other health students [23].

Due to the complex and multifaceted nature of professionalism and e‐professionalism many of the paper's themes overlapped. Nonetheless, we have mapped the 13 manuscripts across five common themes which we will develop below.

### 3.1 Theme 1‐Confirmed Need for e‐Professionalism to Be Part of Formal Curriculum for All Dental Students From BDS1 to BDS5

There was consensus among the sample that dental students needed explicit formal instruction on how to effectively manage the use of social/business media at an undergraduate/postgraduate level. Digital uses impact on student's professional practice both ethical and regulatory could be career defining and it is crucial that the teaching should be mandatory within the undergraduate dental student curriculum as social/business digital media and AI is now all pervasive [15, 16, 17]. This teaching should provide students with a clear guidance of what behaviours using digital platforms constitute professional violations [22] and aligned to the learning objectives/behaviours, learning outcomes and assessment for professionalism in the undergraduate curriculum [13]. Digital professionalism awareness training with practical activities is outlined [12] and training should assist students to manage the risk of inappropriate online presence [14] with emphasis throughout the dental programme on the need for privacy settings however also the reality of privacy settings offering limited protection of data shared and to guard/self‐regulate against inappropriate posting online as they could have long‐term consequences for the students' professional career [11].

### 3.2 Theme 2‐Opportunities and Safe Professional Use

The rapid review draws attention to the positives and negatives associated with social media, with recognition that e‐professionalism teaching needs to include both aspects of the debate in order to promote a more rounded understanding of e‐professionalism. These positives include enabling patient‐practitioner communication, information sharing such as noted during online consultation, for example, as well as supporting professional marketing, self‐directed education, collegiate interaction/support and correction of misleading health information. However, students often criticise the current training stating the lack of content on positive uses of social media. There was a perception that most often we identify and dwell on inappropriate online behaviour was focused on, and rarely were examples of appropriate online behaviour provided, furthermore descriptions of acceptable online behaviour were not explicated [19]. This extensive focus on negative behaviour and subjective norms does not foster change in intentions to be digitally present personally and professionally [12]. There are calls for a more ‘balanced’ view to e‐professionalism teaching, with less scaremongering was proposed [12]. This could include positive example of using DSM/AI for patient benefit showing how DSM/AI could be used as a learning platform for professionalism that reflects the authentic experience in a clinical environment [22] and to foster awareness and positive behaviours about e‐professionalism during undergraduate training [13]. This realistic and fair representation of the digital environment will facilitate education of future dentists and dental professionals and prepare them for entering in practice [21] as well as appreciate the ethical tensions that DSM brings to the business of practising dentistry vs. the ethics of healthcare.

### 3.3 Theme 3‐Students as Co‐Creators

There was an emphasis on co‐designing with students on the development of e‐professionalism guidelines and teaching. Integrating trainees as educators and encouraging peer‐to‐peer regulation could assist in e‐professionalism education. This near‐peer teaching approach can provide personal experiences and more of a ‘nonauthoritative’ approach [22] to the teaching of e‐professionalism. However, dental students will have had limited insight into the pressures of being a qualified professional and their appreciation of the complexities of e‐professionalism may generate within a practice if a digital profile of a dentist conflicts with the practice profile, this would require the teaching to have an experienced practitioner support the delivery of the e‐professionalism in the round.

### 3.4 Theme 4‐Reflection

Undergraduate education must provide opportunity for reflection and critical thinking, which are necessary skills for independent dental practitioners to have as they need to recognise inappropriate behaviour and how to address the behaviour in question [22]. It is important to develop positive attitude towards reflection, self‐direction and humane attributes [23]. Consideration should be given to awareness and awareness training to ensure students are supported in DSM/AI use and its impact on their professional standing [14, 15].

### 3.5 Theme 5‐Identity: Personal/Professional

Personal and professional identity construction is a changing concept and DSM has a growing importance in this construction as it offers places where individuals ‘showcase’ their relevant personal and professional experiences. It therefore requires adopting a holistic perspective of professionalism across both private and professional behaviour [18]. Educators must explicitly address professional values, behaviours and identities of undergraduate health students [23] and prepare students to take control of their online identity and craft a persona that represents their professional image [22]. A supportive holistic teaching approach which demonstrates how to safely optimise the use of digital profiles and identity will give the dental student the confidence to make the right professional choices for themselves.

## 4 Discussion

How do we as teachers support the dentists of the future in navigating e‐professionalism as graduates and undergraduates in the context of the regulators proposed changes to the standards and guidance the GDC provide come 2025?

It is interesting to record the academic interest in quantifying e‐professionalism. At first glance, quantification or the production and communication of numbers [24] represent a concern with accuracy of measures and the collection of data. Many commentators would contend that this focus in the literature is warranted because it allows us to categorise students according to their e‐professionalism, as demonstrating ‘good’ or ‘poor’ e‐professionalism. Poor professionalism was identified as student behaviour which had breached current University Guidance on the use of their own or other data and shared material that was inappropriate on a social media platform.

This aligns with the behaviouralist perspective on professionalism advocated by the regulators and demonstrates how this data on e‐professionalism is ostensibly used as instruments of power and control. However, less is known about the other uses this data could have, specifically, to what extent this information can and should also be used to guide best practice or aid in the remediation of those with lapses in their e‐professionalism. These social and practice‐based implications of the social media data are missing from the debate and the literature [24]. For instance, the UK regulator, the General Dental Council has data to evidence breaches of social media guidance; however, this has not been shared by the GDC to inform any debate on the reality of poor professionalism and its impact on general dental practice.

In proposing these recommendations for e‐professionalism, the authors propose broad principles/codes of practice which encourage individuals to use their insights to apply the principles to individual circumstances.

The use of ChatGPT has demonstrated this philosophy, when initially faced with this dilemma education institutions sought at pace to work *with* rather than against this technological advance balancing acceptance that learners were using this to write assignments and assessments coupled with a lack of ability to detect originality of an individual's work. The outcome places the emphasis on the central tenet of honesty and places the responsibility on the learner to ensure appropriate referencing must be used to acknowledge use of AI in assignments.

This approach attempts to safeguard the principle of honesty in this context and supports the development of e‐professionalism in dental practitioners in training especially as most of these advances can be very useful if correctly engaged with.

The studies included in this review highlight students will be using DSM/AI and they do not want their autonomy impinged upon by regulations but rather they want the tools to safely use DSM/AI.

Having drawn on the rapid review the authors identify two key themes in the literature.

### 4.1 Facilitate Professionalism

In the current online presence, we would suggest providing principles to help safeguard the students' and dentists' professional reputation or themselves, through self‐regulation which would be in line with the direction of travel for the profession, the professions’ relationship with the public. E‐professionalism should be held to the same ethical and regulatory principles as any other interaction or engagement dentists have with patients /public. Professional and personal identities are more intertwined in generations who grew up with internet and social digital media, a space in which they want to maintain their personal online presence but are thirsty for tools to do so safely and with self‐efficacy [25]. Current students have a different threshold for what is acceptable or not. They do not see decisions in their private life any concern of the public or patients as long as they maintain professionalism in their work environment. Adopting an approach which supports appropriate social media and digital interface with the public as well as patients would be more effective than a regulatory/punitive approach. Engagement is always more fruitful with students; if you can frame guidance or teaching in a positive and supportive format, the greater the likelihood of students will adopt the DSM key principles.

### 4.2 E‐Identity and e‐Branding

The graduates of the future will have to navigate the commercial pressures and perceived value within the profession of having an e‐presence which has become a part of the application process for some dental posts where the number of followers or influence/popularity has been a factor in their appointment to a post. The Instagram dentists are considered influencers by the public and the profession because they have generated a following that practices hope to use to increase the practice profile as a centre offering the style or type of dentistry meeting the aesthetic expectations of patients/society. The perceived advantage of a dentist with a large following is it will increase patient uptake of treatment in the practice which should increase the practice revenue leading to the perception you need an active DSM account to be successful. This can lead to a perception that generating a following is mandatory part of being a professional and can lead to an ethical pressure to work hard also in building a DSM following to be commercially viable as well as working hard on the clinical care delivered to continue to grow the number of followers and build a brand which is then marketable to the public/patients. What is the impact of the individual dentist brand which may align to a practice then when the dentist wants to move on the brand they created could limit their options in attracting their next post. The reality also is that the brand of the practice may not align fully with all individual dentists within the practice. The question would be if there was a conflict with the brand of the practice and the dentist how could that impact the dentist, their position and their interaction with patients?

There is a need to protect e‐presence/e‐identity and e‐reputation now and in the future. Some practices focus on cosmetic dentistry expect dentists to have followers and grow their own e‐patient base to enhance the profile of the practice. Professional ethics could be put under pressure when professionals are expected to engage with patients for commercial gain. Having an e‐professional identity is an additional practice expectation for the benefit of enhancing the reputation of the practice which is considered part of the benchmark of whether a modern practice is progressive and perceived as successful. Dental Professional Branding is a growth area not just engaging with the public via websites but also generating a professional identity. An e‐identity/brand could further blur the boundary between the professional and personal relationship of the individual dentist and the profession itself.

### 4.3 Duty of Care to Followers

DSM can generate a following of patients to a dental professional on a scale which could not be managed if face‐to‐face dental advice or treatment options were being delivered. To consider as part of the teaching what if any duty of care is owed to the general public or individual follower both ethically and in practice needs to be addressed.

Under the facilitate theme, we should consider the fact that students and staff want a way to ascertain the validity/quality of Dentistry Digital Social Media content online. We do need a parallel system to that used in lite reviews to assess studies/articles, etc. Potential even a benchmarking or rating from ADEE for example could help support viewers make sense of what we are seeing. Perhaps a similar framework to the Cochrane's Systematic Review guidance. A bit of a ADEE or ADE stamp of approval sets standards to assess Digital Social Media content. We need a way to differentiate posts from credible organisations or individuals versus posts by influencers.

## 5 Conclusion

This rapid review confirms that academic dentistry is aware of the benefits and challenges associated with social media and that social media presents a real challenge to the enacting of professionalism as well as to the teaching of e‐professionalism. E‐professionalism should be integral to the ethical, legal and regulatory teaching which underpins current professionalism within the practice of dentistry. Discreet guidance and direction are necessary for the students and dentists of the future who will need to rely more on their own discretion in how to practice dentistry. Self‐reliance and a clear view of the principles, legislation and working ethical framework, will assist students and dentists to maintain their reputation and practice professionally either face‐to‐face or digitally with patients. The literature review was a valuable insight into what has been written and the need for further academic consideration of e‐professionalism to assist in structuring the teaching, guidance and facilitation of a confident dental professional. A confident dental professional is the outcome sought from teaching to empower the student/ dentist to make the right decision for their future that of the public and the professions continued advancement through technological change.

The findings of the rapid review suggest the need for a broader evidence base on the role and use of social media among dental students and practitioners, in particular the need for more qualitative and pedagogic research, co‐produced with students. The authors acknowledge that this rapid review was undertaken before the upturn in academic interest in AI (artificial intelligence). This technological development presents a significant opportunity for more research on the impact of digital and artificial technology on dental practices, dental education and professionalism.

## Author Contributions

All authors listed confirm they have: contributed to the conception and design, or/and acquisition of data, or/and analysis and interpretation of data; been involved in drafting the manuscript and revising it critically for important intellectual content; given final approval of the version to be published; and agreed to be accountable for all aspects of the work.

## Ethics Statement

The authors have nothing to report.

## Conflicts of Interest

The authors declare no conflicts of interest.

## Data Availability

The authors have nothing to report.
